# Magnetic Vestibular Stimulation in Subjects with Unilateral Labyrinthine Disorders

**DOI:** 10.3389/fneur.2014.00028

**Published:** 2014-03-13

**Authors:** Bryan K. Ward, Dale C. Roberts, Charles C. Della Santina, John P. Carey, David S. Zee

**Affiliations:** ^1^Department of Otolaryngology – Head and Neck Surgery, Johns Hopkins University School of Medicine, Baltimore, MD, USA; ^2^Department of Neurology, Johns Hopkins University School of Medicine, Baltimore, MD, USA; ^3^Department of Biomedical Engineering, Johns Hopkins University School of Medicine, Baltimore, MD, USA; ^4^Department of Neuroscience, Johns Hopkins University School of Medicine, Baltimore, MD, USA; ^5^Department of Ophthalmology, Johns Hopkins University School of Medicine, Baltimore, MD, USA

**Keywords:** vestibular, magnetic, semicircular canals, Lorentz, magneto-hydrodynamics

## Abstract

We recently discovered that static magnetic fields from high-strength MRI machines induce nystagmus in all normal humans, and that a magneto-hydrodynamic Lorentz force, derived from ionic currents in the endolymph and pushing on the cupula, best explains this effect. Individuals with no labyrinthine function have no nystagmus. The influence of magnetic vestibular stimulation (MVS) in individuals with *unilateral* deficits in labyrinthine function is unknown and may provide insight into the mechanism of MVS. These individuals should experience MVS, but with a different pattern of nystagmus consistent with their unilateral deficit in labyrinthine function. We recorded eye movements in the static magnetic field of a 7 T MRI machine in nine individuals with unilateral labyrinthine hypofunction, as determined by head impulse testing and vestibular-evoked myogenic potentials (VEMP). Eye movements were recorded using infrared video-oculography. Static head positions were varied in pitch with the body supine, and slow-phase eye velocity (SPV) was assessed. All subjects exhibited predominantly horizontal nystagmus after entering the magnet head-first, lying supine. The SPV direction reversed when entering feet-first. Pitching chin-to-chest caused subjects to reach a null point for horizontal SPV. Right unilateral vestibular hypofunction (UVH) subjects developed slow-phase-up nystagmus and left UVH subjects, slow-phase-down nystagmus. Vertical and torsional components were consistent with superior semicircular canal excitation or inhibition, respectively, of the intact ear. These findings provide compelling support for the hypothesis that MVS is a result of a Lorentz force and suggest that the function of individual structures within the labyrinth can be assessed with MVS. As a novel method of comfortable and sustained labyrinthine stimulation, MVS can provide new insights into vestibular physiology and pathophysiology.

## Introduction

Case reports of dizziness in and around high-strength (≥3 T) magnets have prompted investigations into the effects of high-strength magnetic fields on human balance and cognitive function. The influence of strong magnetic fields on vestibular function in rats and mice has been identified by circling behavior after magnetic field exposure, an effect that does not occur after prior labyrinthectomy (1, 2). Roberts et al. showed (1) that *all* normal human subjects examined have horizontal nystagmus while lying in a static magnetic field of 7 T and show the slow-phase velocities (SPV) can be as high as 40°/s; (2) the direction of nystagmus changes with head pitch and with direction of entry into the bore; (3) the effect persists throughout the time in the magnetic field (at least to 25 min, the maximum tested thus far); (4) the effect does not depend on rate of motion into or out of the field; (5) the effect scales with the intensity of the magnet field; and (6) the effect is absent in patients with bilateral vestibular loss (3). This magnetic field-induced nystagmus requires only the presence of a static magnetic field; it is not a result of image acquisition.

The best explanation for these effects of magnetic vestibular stimulation (MVS) is that they are due to a static magneto-hydrodynamic (MHD) force, called the Lorentz force, which occurs in a magnetic field due to the presence of normal ionic currents into hair cells within the ion-rich endolymph of the labyrinth. The MHD force produces a pressure in the endolymph that is sensed by the cupula of the lateral semicircular canal (SCC), producing a horizontal nystagmus (3). Quantitative analysis of this behavior suggests that resting utricular hair cell current is primarily responsible for generating the MHD force (4, 9). Because the utricle is close to the opening of the lateral SCC, the pressure from the Lorentz force it generates can deflect the cupula of the lateral SCC. Individuals with unilateral vestibular hypofunction (UVH) have asymmetric remaining vestibular function and should have a similar MVS response to those with intact function, but proportional to the residual function of their remaining utricle and SCCs. The goal of the present study was to investigate MVS in humans with UVH to explore further the mechanisms involved in MVS and suggest a potential clinical use for MVS in evaluating the function of individual structures within the labyrinth.

## Materials and Methods

Nine subjects with UVH were studied. Subjects lay supine in a Philips Achieva 7 T MRI magnetic field (Philips Research, Hamburg, Germany) for trials up to 5 min. Eye movements were recorded in darkness using infrared video-oculography (VOG, horizontal and vertical) captured at 30 frames/s with 640 × 480 resolution (Resonance Technology, Inc., Los Angeles, CA) while the subjects remained still. No MRI images were taken during the study. Torsional eye movements, which were small, were assessed qualitatively from the video images.

Subjects were first placed supine on the MRI’s table with their heads near the bore in a neutral neck flexion/extension position (neutral) as if for an MRI head scan. The pitch angle was measured using a non-metallic protractor with a bubble level. The external reference was taken as the line from the lateral canthus of the eye to the tragus, approximating Reid’s horizontal plane such that the lateral SCCs are angled about 20° upward from this line (4). Before each subsequent entry into the magnetic field bore, the angle of the subject’s head pitch was altered and measured.

Eye movements were recorded from the subject’s right eye using infrared VOG. For two subjects, a second infrared camera recorded movements of the other eye to see if the eye movements were conjugate. Subjects’ eye movements were calibrated outside the magnetic field while supine with the head neutral on the table (not pitched up or down) and looking directly at a target screen above. The VOG goggles remained fixed on the head throughout collection of all data files. Calibration was repeated whenever the goggles were removed or repositioned.

After calibration, the room lights were turned off and vision was prevented by covering the subject’s head with a double layer of black felt cloth. After baseline eye movement recordings were taken outside the bore, a subject was moved into the bore using the fixed-speed table motor drive (10.8 cm/s over 2 m travel). The field strength outside the bore near the subject’s ears was approximately 0.7 T.

For each subject, at least four trials were performed entering into the magnet head-first. For each trial, the subject’s head was positioned at a different pitch angle by placing pads under the subject’s neck and shoulders or at the back of the head. For five subjects, there was one additional recording with feet-first into the magnetic field, in the supine position at the approximate original head pitch angle. All subjects additionally had eye movements recorded while sitting and lying supine in a room away from the field of the magnet.

The direction of the magnetic field vector *B* was directed from the subject’s head toward the feet when entering the magnet supine and head-first, i.e., in the −Z direction when expressed in the RAS radiological coordinate system [+X/right, +Y/anterior, +Z/superior]. Two sensors monitored the magnetic field near the head: a gauss meter (AlphaLabs/Trifield GM-2, range up to 3 T) measured absolute field strength, and a custom-built wire coil (75 turns of AWG36 magnet wire on 12 mm circular frame) measured change in magnetic field over time (d*B*/dt) as the table moved into the bore.

Times of entry into and exit from the magnetic field were recorded by placing the wire coil near the subject’s head and recording changes in field strength over time. Data were collected using a custom-written computer program (using Microsoft Visual C++). Eye movements and analog sensor data were synchronized for later analysis using MatLab (MathWorks, Natick, MA).

### Study population

Study subjects were recruited from the clinical practices of the investigators and all subjects consented to this research according to a protocol approved by the institutional review board. Subjects were diagnosed with UVH based on history and physical examination and physiological data including vestibular-evoked myogenic potentials (VEMP) and quantitative head impulse testing.

Subject age ranged from 29 to 65 with mean age (standard deviation, SD) of 53.8 (11.6) years. There were three men and six women. Five subjects had left-sided UVH and four right-sided. Table 1 shows demographic and physiologic data for all subjects. Quantitative head impulse testing was performed in eight subjects and VEMP data were available in nine. In darkness, without fixation, and away from the magnetic field, all subjects demonstrated a low-velocity spontaneous nystagmus with slow-phases toward the affected labyrinth, consistent with a nearly completely compensated unilateral vestibular deficit.

**Table 1:** **Demographics and physiological findings**.

Subject	Age	Side	Diagnosis, cause of unilateral loss	Duration (months)	Baseline SPV, in darkness (°/s)	HIT gain						oVEMP		cVEMP	
						L-SCC		S-SCC		P-SCC	
						Left	Right	Left	Right	Left	Right	Left	Right	Left	Right
1^a^	64	L	Sub-occipital approach for schwannoma resection	28	2.6 left, 1.3 down	NA	NA	NA	NA	NA	NA	NA	NA	NA	NA
2	65	L	Sub-occipital approach for schwannoma resection	19	1.3 left, 0.1 down	0.64	0.86	0.40	0.96	0.41	0.89	Absent	Intact	Absent	Absent
3	58	L	Probable vestibular neuritis	13	0.1 left, 3.4 down	0.26	0.90	0.30	1.2	1.09	1.0	Absent	Absent	Absent	Reduced
4	29	L	Cholesteatoma with SCC fistula	5	2.7 left, 0.7 down	0.52	0.82	0.21	0.70	1.12	1.05	Reduced	Intact	Reduced	Intact
5	51	L	Sub-occipital approach for schwannoma resection	5	0.6 left	0.33	0.69	0.17	0.91	0.19	1.0	NA	NA	NA	NA

6	53	R	Labyrinthectomy for Meniere’s disease	2	1.3 right, 0.2 down	0.82	0.44	1.01	0.42	0.91	0.46	Intact	Absent	Intact	Absent
7	58	R	Trans-labyrinthine approach for schwannoma resection	300	1.0 right, 0.1 up	0.96	0.58	NA	NA	NA	NA	Intact	Absent	Intact	Absent
8	46	R	Sub-occipital approach for schwannoma resection	2	1.7 right, 4.8 down	0.67	0.17	NA	NA	NA	NA	Intact	Absent	Intact	Absent
9	65	R	Gentamicin injection and vestibular nerve section for Meniere’s disease	72	2.3 right, 2.7 down	0.73	0.23	0.82	0.63	0.63	0.31	Intact	Absent	Intact	Absent

*^a^Subject 1 demonstrated overt corrective saccades for left LSC (clinical exam)*.

*Head impulse tests (HIT) were recorded using wire coils in six and video goggles in two subjects (subjects 7 and 8) and qualitatively assessed in one (subject 1). Ocular vestibular-evoked myogenic potential (oVEMP) performed with air-conducted clicks and reflex hammer taps unless otherwise specified and cervical vestibular-evoked myogenic potential (cVEMP) performed with air-conducted clicks. SP, slow-phase; L-SSC, lateral SCC; S-SSC, superior SCC; P-SSC, posterior SCC; NA, not available; TB, tone burst. Gray shading indicates relatively weak labyrinth*.

Cervical VEMPs (cVEMP) and ocular VEMPs (oVEMP) were elicited with click stimuli as previously described (5). The cVEMP p13–n23 response and oVEMP n10 amplitude was used to assess saccular and utricular function, respectively. Angular vestibulo-ocular reflex was quantified in all subjects but one with head impulses in the planes of each of the SCCs using either video goggles recording at a 300-Hz frame rate or search coils as previously described (6). Subjects focused on a point target 1 m away while rapid, small excursion, high-acceleration head impulses were imposed. Gain <0.68 was used to indicate SCC hypofunction (7). Video goggles were calibrated only for assessing lateral SCC gain.

### Data analysis

Pupil tracking software (ViewPoint Eye Tracking, Arrington Research Inc., Scottsdale) saved normalized pupil positions in the data file for each trial. Custom-written MatLab programs analyzed the horizontal and vertical components of eye movement and d*B*/dt data. Known target locations were used to calibrate eye positions in degrees. Nystagmus was manually marked near the beginning and end of each slow-phase, and a least-squares line was automatically fitted to the data between the marked points. The slope of each slow-phase line became a single slow-phase velocity data point. The torsional component of nystagmus was usually quite small and was assessed qualitatively by eye.

Mean slow-phase velocity was calculated for each trial over a 40-s time interval once inside the MRI bore. Baseline slow-phase velocity outside the magnet was subtracted from that in the magnet to get the change in mean SPV. Differences between subjects in slow-phase velocity for head- and feet-first paradigms were assessed using Wilcoxon rank-sum test. Medians and interquartile ranges (IQR) are therefore presented for between-group comparisons. Associations were considered statistically significant for two-sided statistics with a *P* value <0.05. All analyses were performed using Stata 12.0 (StataCorp, College Station, TX, USA).

## Results

**Figure 1:**
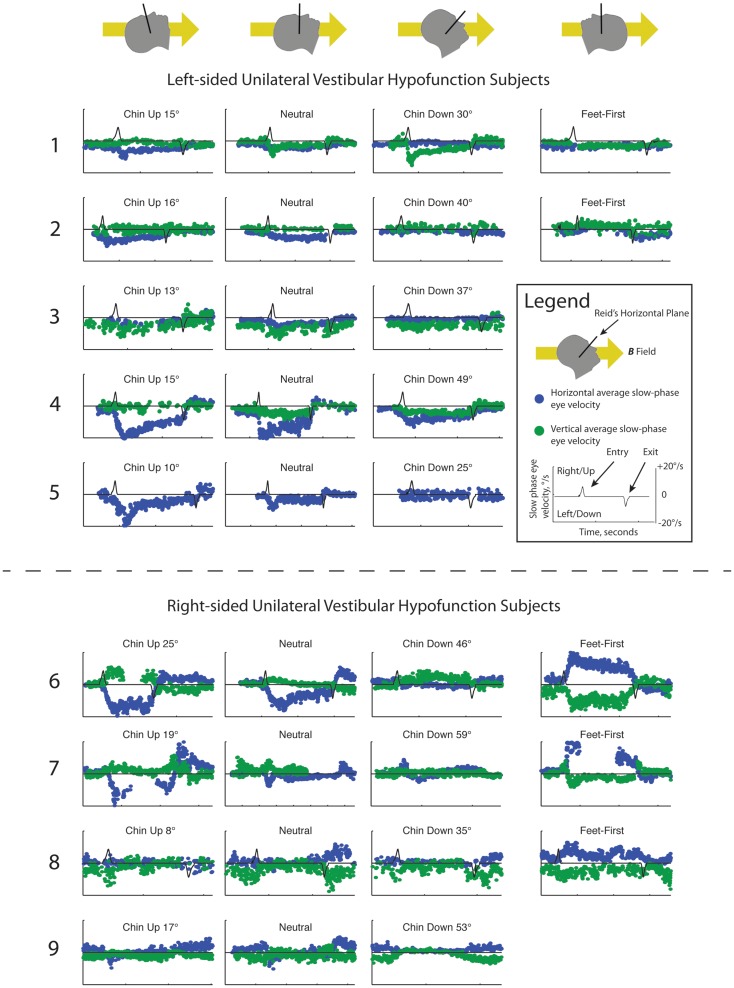
**Slow-phase nystagmus eye velocity over time for all subjects**. Each column represents a separate trial with the subject’s head pitched either chin up or chin down from a head-neutral supine position. The last column represents feet-first in subjects in which this data was available. Horizontal slow-phase velocity is shown with blue dots and vertical slow-phase velocity with green dots. Solid black line represents electromagnetic induction (d*B*/dt), measured using a wire coil. Electromagnetic induction peak and trough indicate, respectively, entry and exit from the bore of the magnet.

Slow-phase velocities for all subjects are shown in Figure 1. Subjects entered the magnetic field with a mean neutral head angle of 116°(SD 7.1°) relative to Earth horizontal. While supine in the neutral head position, median absolute change in SPV from outside the bore to inside the 7 T magnetic field was 4.6°/s (IQR 2.3–6.2) for the horizontal component and 2.8°/s (IQR 1.3–3.2) for the vertical component of nystagmus (*P* = 0.28). Across all subjects, the peak absolute change in horizontal SPV (6.1°/s, IQR 4.6–12.2) was greater than the peak vertical component (3.9°/s, IQR 2.8–4.6, *P* = 0.03). There was no difference in change in SPV between right- and left-sided unilateral vestibular loss subjects for the horizontal component of nystagmus (*P* = 0.62), with all UVH subjects demonstrating a nystagmus with leftward slow-phases on entering the magnetic field at the neutral, lying flat head position or with their heads pitched chin up. However, there was a striking difference in mean change in SPV of the vertical component between right- and left-sided UVH subjects in the magnetic field: right-sided UVH subjects developed upward slow-phases and left-sided UVH subjects developed downward slow-phases (*P* < 0.001). Torsional responses were small and sometimes imperceptible but when present their pattern was always consonant with the change in vertical direction (discussed below).

For the five subjects who were also tested feet-first in the neutral head position, the direction of horizontal nystagmus reversed to slow-phase right in four subjects (Subjects 2, 6–8) and decreased to a null in one left-sided UVH subject (Subject 1). The median absolute change in SPV from outside the magnet to after entering the 7 T magnetic field feet-first was 4.9°/s (IQR 1.8–11.6) for the horizontal component and 2.2°/s (IQR 0.2–2.4) for the vertical component, which were not different from the absolute changes recorded with head-first entry at approximately the same pitch angle (*P* > 0.05). Three of five subjects also showed a reversal of the vertical component when entering the magnetic field feet-first (right-sided UVH subjects 6, 7, and 8), whereas left-sided UVH subject 1 decreased their downward SPV toward a null and subject 2 developed upward slow-phases.

**Figure 2:**
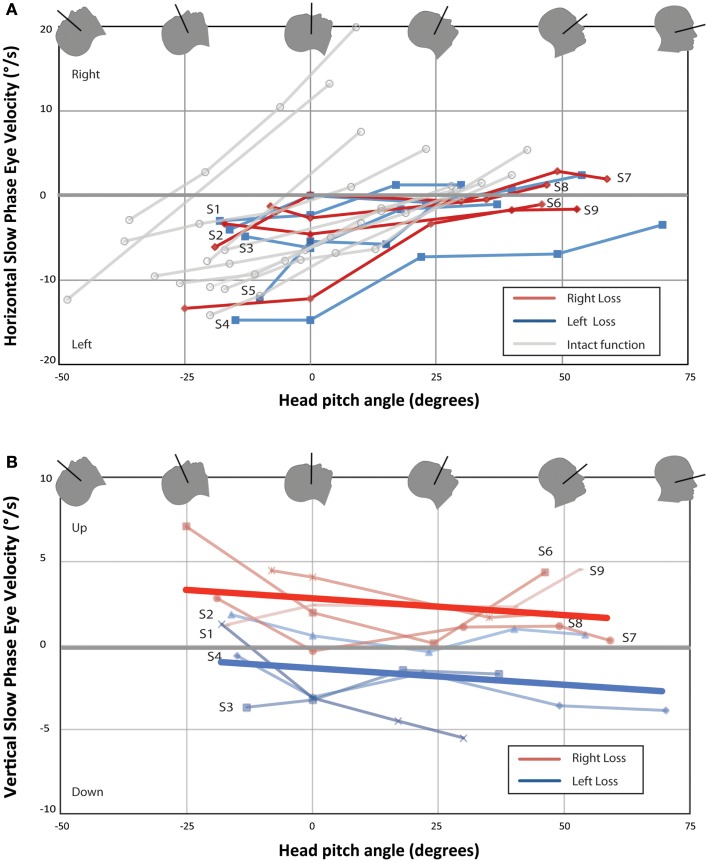
**Change in nystagmus slow-phase horizontal (A) and vertical (B) eye velocity from baseline eye velocity for all UVH subjects**. Each point represents the difference in average SPV between a 40-s time interval once the subject has entered the bore and baseline average SPV in darkness prior to entry. Right-sided UVH subjects are labeled to the right and left-sided UVH subjects to the left of each line. Ten subjects with bilateral intact vestibular function from Roberts et al. are plotted in **(A)** for comparison (3). Lines of best-fit (linear least-squares method) are shown in **(B)** for right-sided UVH and left-sided UVH. **(A)** All subjects developed a slow-phase left nystagmus that decreased in velocity with chin down head pitch, with four subjects demonstrating reversal of horizontal nystagmus direction with head pitch beyond a null position. **(B)** Right-sided loss subjects developed slow-phase-up nystagmus inside the magnetic field and left-sided loss subjects developed slow-phase-down nystagmus.

Figure 2 shows mean horizontal and vertical SPV as functions of head pitch angle for each UVH subject and horizontal SPV for 10 subjects with bilateral intact vestibular function previously reported (3). Eight of nine UVH subjects showed a null position for the horizontal component of nystagmus (Figure 2A). Subjects 1, 2, and 7 also showed a reversal of the direction of the horizontal component of nystagmus with pitching the chin down beyond the null position. The slopes of best-fit lines for each subject for horizontal SPV vs. pitch angle was significantly larger for subjects with intact vestibular function than for UVH subjects [median 0.26 (IQR 0.18–0.49) vs. 0.10 (IQR 0.07–0.14), *P* < 0.01]. Median peak leftward SPV in subjects with intact vestibular function was about twice that of UVH subjects, but in our sample size this was not significantly different (11.0°/s, IQR 7.5–12.0 vs. 6.1°/s, IQR 4.1–12.2, *P* = 0.16). In Figure 2B, one sees the effect of head pitch on vertical SPV. The main findings are that the left-sided UVH almost always remain above (positive, upward) relative to the right UVH, and the slope changes little with head position. In both subjects in whom binocular eye movements were recorded, eye movements during the nystagmus response were qualitatively conjugate.

## Discussion

When lying in a 7 T MRI machine, subjects with UVH develop a characteristic pattern of vertical (and torsional) nystagmus that differs from the purely horizontal nystagmus shown by subjects with intact labyrinthine function (3). While patients with UVH have a pattern of horizontal nystagmus similar to normal subjects, they also show a striking vertical component with a direction that depends upon which labyrinth is intact. Right UVH patients have a slow-phase-up component and left UVH subjects a slow-phase-down component.

**Figure 3:**
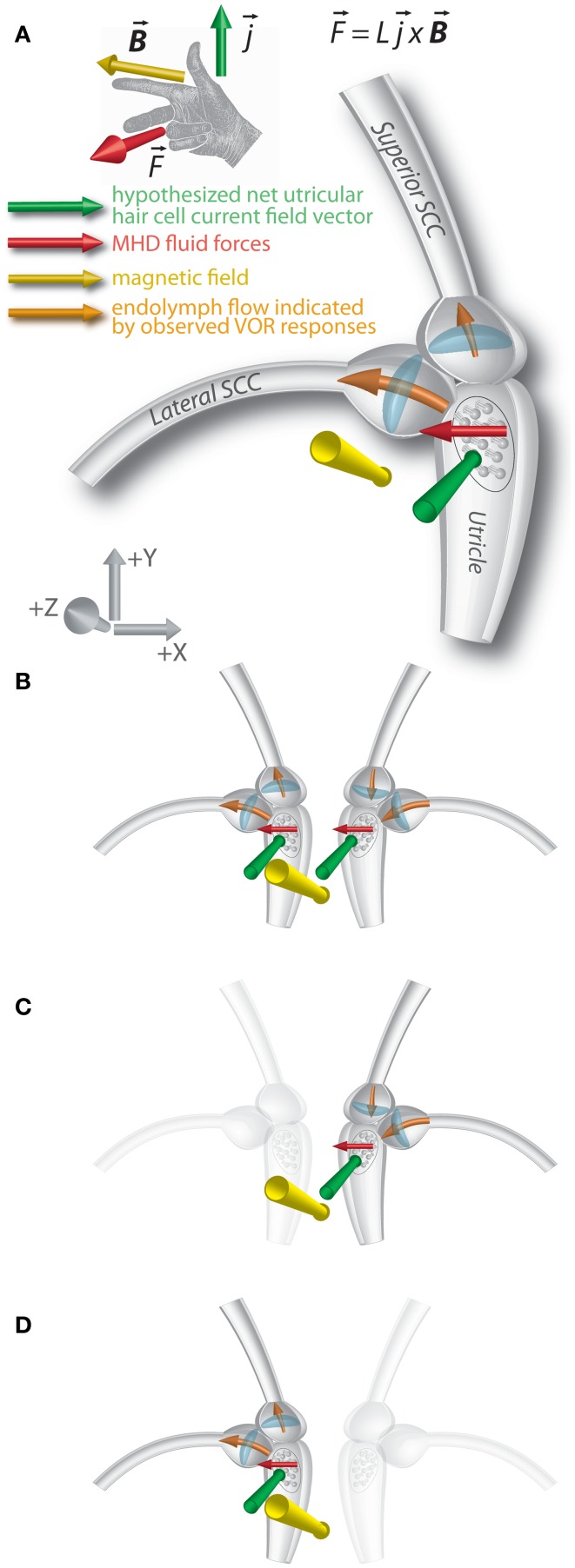
**Lorentz force vector diagram of magnetic vestibular stimulation (MVS)**. **(A)** An ionic current in a magnetic field results in a magneto-hydrodynamic (MHD) force $\left(\vec{F}\right),$ represented by the cross product of the current $\left(\vec{j}\right)$ and magnetic field vectors $\left(\vec{B}\right).$ *L* represents the scalar length over which the current flows. A right-hand rule demonstrates this relationship. The MHD force induces endolymph flow, which deflects the horizontal and superior canal cupulae. Axis represents RAS radiological coordinate system [+X/right, +Y/anterior, +Z/superior]. The images show the magnetic field vector in the −Z orientation. **(B)** In individuals with intact vestibular function on both sides, effects of MHD stimulation in the right and left lateral canal cupulae sum, and horizontal nystagmus is observed. The forces on the superior canal cupulae are inhibitory on the right and excitatory on left, so no vertical eye movements are observed. **(C)** In those with left-sided loss, the force on the right superior canal cupula is inhibitory, and downward slow-phases are observed in the magnetic field. **(D)** In those with right-sided loss, the force on the left superior canal cupula is excitatory and upward slow phases are observed in the magnetic field.

We previously hypothesized that the nystagmus in the magnetic field shown by normal subjects is due to a static MHD force – the Lorentz force – produced by ionic currents in the endolymph of the labyrinth (3). The force has been attributed primarily to the ionic currents associated with the utricle, though the effect would not be on the utricle macula and hair cells themselves, but through the movement of the endolymph into the nearby opening of the lateral SCC, pushing on the cupula and producing a nystagmus (Figure 3A). Although ionic currents in the cristae of the SCCs could also generate an MHD force (3, 8), the current density would be less than in the utricle due to fewer hair cells (9, 10), resulting in a weaker MHD Lorentz force. The saccule, too, would generate an MHD force, but is anatomically isolated from the SCCs.

Given how close the openings of the lateral and superior SCCs are to the region that should be affected by MHD-generated endolymph currents driven by the utricle, the cupulae of both canals could be displaced in a sufficiently strong magnetic field. For the lateral SCC, the MHD force would excite the right lateral SCC and inhibit the left, similar to a head turn to the right (Figure 3B). With their head neutral or pitched chin up, UVH subjects developed a nystagmus with leftward slow-phases in the magnetic field when entering head-first, regardless of the side of their loss of function. For those with left-sided vestibular loss, this suggests the cupula from their right (intact) lateral canal is deflected in an excitatory direction (ampullopetal) in the magnetic field; for right-sided vestibular loss, the nystagmus with leftward slow-phases suggests their left lateral canal cupula is deflected in an inhibitory direction (ampullofugal). In either case, the nystagmus would have leftward slow-phases. The sensitivity of peak SPV to head pitch for UVH subjects was also decreased, to approximately half that of subjects with intact vestibular function and this difference was statistically significant (Figure 2A). This finding is consistent with the MVS hypothesis.

Similar to subjects with intact vestibular function, UVH subjects have a head pitch null position where no horizontal nystagmus is seen. This null position varies from subject-to-subject (Figure 2A), but as in the majority of subjects with intact vestibular function, it was close to the orientation at which the lateral SCC plane is perpendicular to the magnetic field vector (Reid’s plane pitched chin down approximately 20°). In this head position, the mean putative utricular current field vector and magnetic field vectors are approximately aligned, producing no net Lorentz force. Three subjects (subject 1, 2, and 7) reversed the direction of the horizontal component with the head pitched chin down beyond their null position. In addition, all subjects either reversed direction of the horizontal component of nystagmus or were at a null when entering the bore feet-first, confirming that the direction of nystagmus depends on head orientation with respect to the orientation and polarity of the magnetic field orientation.

In our prior study, subjects with either bilateral intact or bilateral absent vestibular function showed no vertical or torsion eye movements (3). In contrast, all UVH subjects exhibited a vertical component of nystagmus. Despite the direction of horizontal nystagmus being the same in all subjects, those with left-sided UVH developed a slow-phase-down component inside the magnetic field, with three subjects reversing to slow-phase-up on exit from the bore. Right-sided UVH subjects, however, developed a slow-phase-up nystagmus in the bore, with two subjects reversing to slow-phase-down on exit from the bore. Although the vertical component of the nystagmus was relatively small, the differences in the patterns of the right and left UVH subjects were consistent in all subjects and suggest differential excitation or inhibition, respectively, of the intact vertical SCC.

We propose that this asymmetry of the vertical component reflects, in those with left-sided vestibular loss, *inhibition* of the right superior SCC (ampullopetal flow, resulting in a slow-phase-down component, Figure 3C), and, in those with right-sided loss, *excitation* of the left superior SCC (ampullofugal flow, resulting in a slow-phase-up component, Figure 3D). Qualitative assessment of torsion was also compatible with this hypothesis (discussed below). For those with bilateral intact vestibular function, an MHD force driven by an anteroinferiorly directed net utricular current field would deflect the right superior SCC cupula in an inhibitory direction and the left in an excitatory direction, resulting in cancelation of the vertical components of nystagmus (Figure 3B). Although torsion components of the superior SCCs should sum, the lateral SCC contribution also drives a torsion component due to its pitched-up orientation when the *B* field is perpendicular to Reid’s horizontal plane (4, 11), potentially canceling the torsion due to excitation of the left superior SCC or inhibition of the right superior SCC.

The superior SCC cristae can be seen using three-dimensional surface rendering of sectioned temporal bones (12). The saddle-shaped crista of the superior SCC forms the seat of the SCC cupula and is oriented so that its longest dimension (from “front” to “back” of the saddle) lies in a plane perpendicular to the plane of the SCC and offset about 30° from the mean plane of the utricular macula. This relationship between the utricle and lateral/superior SCC cupulae would allow generation of an MHD force that differentially excites or inhibits the remaining superior SCC in individuals with unilateral vestibular loss.

Torsional eye movements were small and could only be qualitatively assessed with the MRI-compatible VOG system we used. This may in part be due to the larger horizontal and vertical components creating noise in the relatively small torsion signal. When present, torsional eye movements nevertheless always fit the pattern of labyrinthine stimulation inferred from the directions of the vertical nystagmus components. Four of five left-sided loss subjects exhibited small torsional eye movements when exiting the magnet bore. In these cases, slow-phases included both an upward component and torsion that would be counter-clockwise from the subject’s frame of reference. If the superior and lateral SCC were functioning only on the right side in these subjects, the direction of the torsional and vertical component observed while exiting the bore is compatible with the right superior SCC having been released from *inhibition* that occurred while in the bore. Left-sided UVH subject 1 demonstrated a strong counter-clockwise torsion component inside the magnet in the *feet-first* position, in which case the direction of the MHD force relative to the labyrinth is reversed. This finding implies *excitation* of the right superior canal while in the magnet feet-first. Three of four right-sided loss subjects qualitatively demonstrated a small clockwise torsion component while inside the magnet, compatible with the intact left superior SCC having been *excited*. No torsion was observed immediately after exiting the magnet or on feet-first entry for the right-sided UVH subjects. Taken together, the pattern of vertical and torsional eye movements in all UVH subjects, both in and outside the magnet and with a head-first or feet-first orientation in the bore suggests the induced eye movements are the result of excitation in the intact left superior SCC in right UVH subjects or inhibition in the intact right superior SCC of left UVH subjects.

## Conclusion

The pattern of horizontal and vertical nystagmus induced in subjects with UVH is consistent with the Lorentz force hypothesis for activation of the labyrinths in the static magnetic fields of MRI machines. The horizontal component of nystagmus shown by subjects with intact labyrinthine function and UVH subjects is consistent with excitation of the lateral SCC on one side and inhibition on the other, and it depends on head orientation in the bore with respect to the polarity of the magnetic field. The vertical (and torsional) component of the nystagmus, so far seen only in UVH subjects, reflects excitation or inhibition of the superior SCC in the intact labyrinth, depending on the side of the intact labyrinth and on the orientation of the head in the magnetic field. Our results further suggest a use for MVS in evaluating the function of individual labyrinthine structures as well as a novel and comfortable way to induce a sustained nystagmus in normal subjects and study the response of adaptive mechanisms to a pathological vestibular imbalance.

## Conflict of Interest Statement

The authors declare that the research was conducted in the absence of any commercial or financial relationships that could be construed as a potential conflict of interest.
